# Angle-dependent photodegradation over ZnO nanowire arrays on flexible paper substrates

**DOI:** 10.1186/1556-276X-9-667

**Published:** 2014-12-11

**Authors:** Ming-Yen Lu, Yen-Ti Tseng, Cheng-Yao Chiu

**Affiliations:** 1 Graduate Institute of Opto-Mechatronics, National Chung Cheng University, Chia-Yi 62102, Taiwan; 2 Advanced Institute of Manufacturing with High-tech Innovations, National Chung Cheng University, Chia-Yi 62102, Taiwan

**Keywords:** Photodegradation, ZnO nanowire arrays, Paper substrates, Angle-dependent

## Abstract

In this study, we grew zinc oxide (ZnO) nanowire arrays on paper substrates using a two-step growth strategy. In the first step, we formed single-crystalline ZnO nanoparticles of uniform size distribution (ca. 4 nm) as seeds for the hydrothermal growth of the ZnO nanowire arrays. After spin-coating of these seeds onto paper, we grew ZnO nanowire arrays conformally on these substrates. The crystal structure of a ZnO nanowire revealed that the nanowires were single-crystalline and had grown along the *c* axis. Further visualization through annular bright field scanning transmission electron microscopy revealed that the hydrothermally grown ZnO nanowires possessed Zn polarity. From photocatalytic activity measurements of the ZnO nanowire (NW) arrays on paper substrate, we extracted rate constants of 0.415, 0.244, 0.195, and 0.08 s^-1^ for the degradation of methylene blue at incident angles of 0°, 30°, 60°, and 75°, respectively; that is, the photocatalytic activity of these ZnO nanowire arrays was related to the cosine of the incident angle of the UV light. Accordingly, these materials have promising applications in the design of sterilization systems and light-harvesting devices.

## Background

The integration of inorganic nanomaterials into soft substrates enables the development of flexible functional devices, including rollup displays, electronic paper, and wearable devices. When using paper or paper-like substrates, such systems have a diverse range of possible applications in photodetectors [1], light-emitting diodes [2], piezoelectric nanogenerators [3], paper batteries [4], and electronic paper displays [5]. Paper-like substrates are cheap, light, flexible, and biocompatible; accordingly, innovative techniques and strategies based on paper-like platforms are just beginning to emerge from laboratory-level studies.

Because of their wide direct band gap (*E*_g_ = 3.37 eV) and large exciton energy (60 meV), zinc oxide (ZnO) nanostructures have diverse applications in light-emitting diodes [6], solar cells [7], gas sensors [8], photodetectors [9], and nanogenerators [10,11]. In addition to high-temperature vapor transport growth [12], low-temperature hydrothermal growth is a facile means of growing ZnO nanowire (NW) arrays of high crystal quality; this method is not only feasible for tuning the semiconductor properties by doping during the growth process [13] but also has the flexibility to allow the growth of NWs on a variety of substances (e.g., polymers [14], cotton fibers [15], paper [1], wood [16]). The growth of uniform NW arrays is relatively difficult to achieve on substrates that have high surface roughness. ZnO nanostructures are among the most promising materials for practical photocatalysts that can be used for the removal of organic or toxic pollutants [17], sterilization [18], and water splitting [19]. The degradation of dyes is a common means of gauging the efficiency for photocatalysis [20]. We are unaware, however, of any previous reports of the incident angle-dependence of the photodegradation properties of ZnO NW arrays.

Herein, we report a facile strategy for the conformal growth of ZnO NW arrays on paper substrates; the uniformity of these ZnO NW arrays was affected by the ZnO seeding conditions. In addition, we have observed incident angle-dependence of the photodegradation properties of these ZnO NW arrays on paper substrates.

## Methods

The growth of ZnO NW arrays on paper substrates was performed using a two-step growth strategy, as previously described [16]. The ZnO nanoparticles (NPs), used as seeds in this study, were formed by adding a solution of 50 mM NaOH in EtOH (5, 10, 15, 25, 35, or 45 mL) into a solution of 50 mM zinc acetate [Zn(CH_3_COO)_2_ · 2H_2_O] in EtOH (25 mL) and then stirring the mixed solution at 60°C for 2 h; the color of the solution changed from white to colorless, indicating the formation of ZnO NPs. The conditions are listed in Table 1. The ZnO NPs were then spun on paper substrates and dried in an oven at 80°C for a few hours to improve their adhesion. The paper we used is bought from the commercial merchandise, which mainly contains cellulose, CaCO_3_, and binders. The scanning electron microscopy (SEM) image and energy-dispersive X-ray spectroscopy (EDS) spectrum of bare paper substrate is shown in Additional file 1: Figure S1. The papers’ surface is rough because of cellulose fibers; and C, Ca, and O signals in the EDS spectrum confirm the existence of cellulose and CaCO_3_. The Pt signal in the EDS spectrum is from the thin Pt conducting layer we deposited to improve the image quality in SEM. The growth of ZnO NW arrays was achieved by immersing the seeded paper substrates in the growth solution at 90°C for 12 h; the growth solution comprised equal amounts of 25 mM Zn(NO_3_)_2_ and 25 mM hexamethylenetetramine (HMTA). After growth, the samples were rinsed with deionized water and then dried in an oven.

**Table 1:** Recipes for synthesizing ZnO NPs

**ZnO nanoparticle solutions**	**A**	**B**	**C**	**D**	**E**	**F**
50 mM Zinc acetate (mL)	25	25	25	25	25	25
50 mM NaOH (mL)	5	10	15	25	35	45

The morphologies of samples were analyzed using SEM. The crystal structures and the compositions of the ZnO NPs and ZnO NWs were characterized through X-ray diffractometry (XRD; Bruker D2 phaser, Bruker AXS, Inc., Madison, WI, USA) and transmission electron microscopy (TEM; JEOL ARM 200 F, JEOL Ltd., Akishima-shi, Japan); the transmission electron microscope could also be employed for scanning transmission electron microscopy (STEM) under aberration-corrected conditions. The photocatalytic activities of the ZnO NWs on paper substrates were examined through their ability to degrade a solution of methylene blue (MB) at room temperature in the homemade illumination setup. A sample of ZnO NW arrays on paper substrate (1 cm^2^) was immersed in a test bottle containing 114 μM MB (16 mL). A 13-W UV lamp operating at a wavelength of 365 nm was used as a radiation light source; it was placed 15 cm from the test bottle. The solution was stirred continuously during the irradiation procedure. The concentration of MB was monitored using a UV-vis spectrophotometer; aliquots (2 mL) of the MB solution were removed at various reaction periods for UV-vis spectroscopic analyses.

## Results and discussion

In general, a ZnO-seeded substrate is required to form uniform ZnO nanowire/nanorod arrays through hydrothermal growth [21]. The most common way to prepare a seeded substrate is to spin-cast a solution of zinc ions onto the substrate and then to oxidize the sample at a temperature higher than 300°C to form ZnO seeds. Because paper and plastic substrates might not be stable at high temperatures, we did not wish to use this seeding method in this present study. Therefore, we employed ZnO NPs as seeds for hydrothermal growth to avoid high-temperature processing.

**Figure 1:**
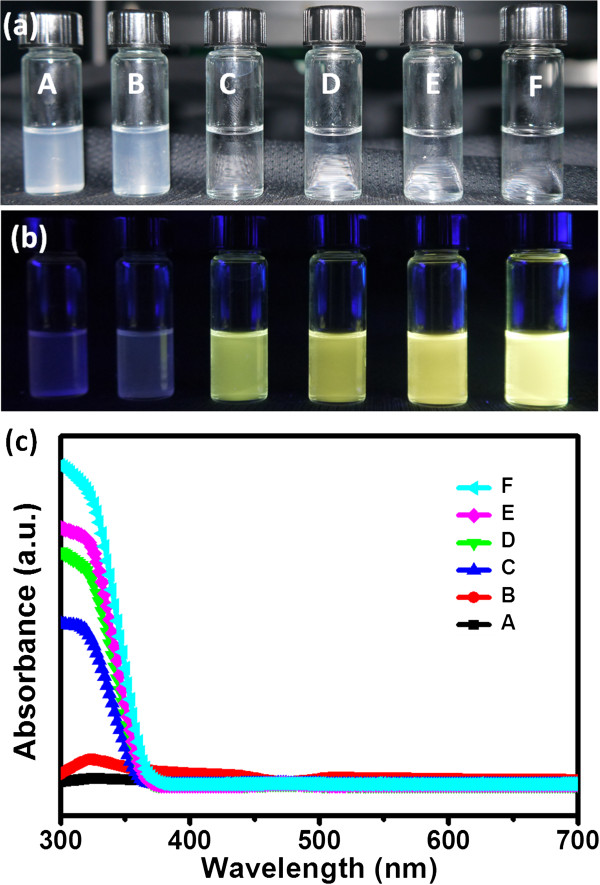
**Photographs and absorption spectra of ZnO NP solutions. (a, b)** Photographs of ZnO NP solutions prepared using different recipes in the **(a)** absence and **(b)** presence of UV irradiation. **(c)** Absorption spectra of these ZnO NP solutions.

Figure 1 displays photographs and absorption spectra of ZnO NP solutions obtained after adding the various amounts (5, 10, 15, 25, 35, and 45 mL) of 50 mM NaOH in EtOH to 50 mM Zn(OAc)_2_ in EtOH (25 mL) and then heating at 60°C for 2 h. The solutions (A to F, respectively) gradually turned colorless, indicating a greater degree of ZnO NP formation, upon increasing the amount of added NaOH solution (Figure 1a). The ZnO NP solutions emitted light under excitation with UV light (Figure 1b), with the intensity of the emitted light increasing with respect to the amount of NaOH that had been added into the solution. The yellow emission of the ZnO NPs presumably resulted from defects in the ZnO [22]. The ZnO nanoparticle formation is influenced by the pH values of solution, which is controlled by the amount of NaOH we added. The pH values are plotted in Additional file 1: Figure S2; the pH values increased with the adding amount of NaOH. The absorption spectra of the ZnO NP solutions (Figure 1c) reveal that the absorption edge of the ZnO NPs appeared near 380 nm, with the absorption increasing with respect to the amounts of added NaOH; a stronger absorption implied a higher concentration of ZnO NPs in the solution.

**Figure 2:**
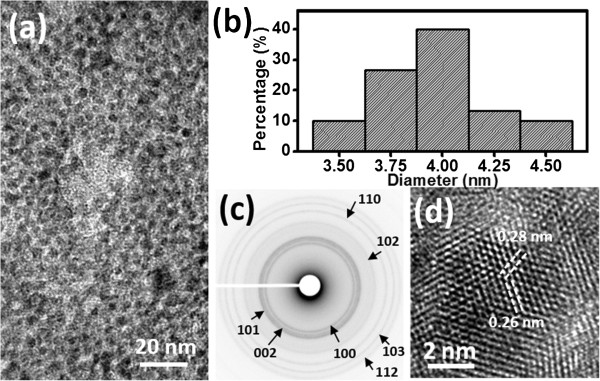
**TEM analysis of ZnO NPs. (a)** Low**-**magnification TEM image of ZnO NPs synthesized from 25 mL of 50 mM Zn(OAc)_2_ and 45 mL of 50 mM NaOH. **(b)** Size distribution of the ZnO NPs obtained from (a); average size of the NPs: ca. 4 nm. **(c)** SAD pattern of ZnO NPs; all the rings are indexed to wurtzite-ZnO. **(d)** High-resolution TEM image of a single ZnO NP; the spacings of 0.26 and 0.28 nm correspond to the (002) and (100) planes of ZnO, respectively.

The low-magnification TEM image in Figure 2 reveals that the ZnO NPs had sphere-like shapes and a uniform size distribution; the ZnO NPs had diameters in the range 3 to 5 nm with an average size of approximately 4 nm (Figure 2b). In the corresponding selected area diffraction (SAD) pattern of the ZnO NPs (Figure 2c), the ring figure was contributed by a number of ZnO NPs in Figure 2a, with all of the rings indexed to wurtzite-structured ZnO. Figure 2d presents the high-resolution (HR) TEM image of a single ZnO NP. The *d*-spacings of 0.26 and 0.28 nm correspond to the (002) and (100) planes of ZnO, respectively; the successive atomic arrangements of a single NP indicate the single-crystalline nature of these ZnO NPs. Next, we spun a solution of the prepared ZnO NPs onto paper substrates; we observed better coverage of the ZnO NW arrays on the paper substrate when using the ZnO NPs from recipe F (Additional file 1: Figure S3).

**Figure 3:**
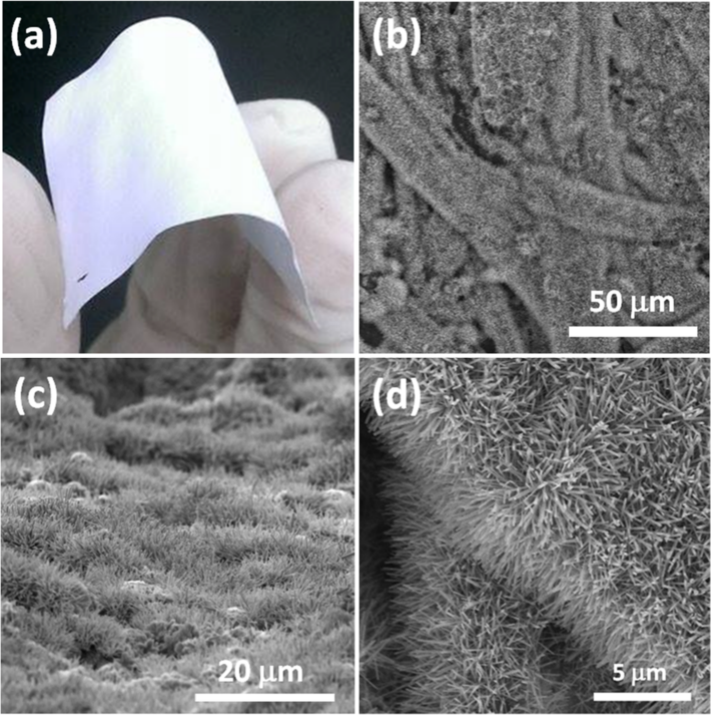
**Photograph and SEM images of ZnO NW arrays on a paper substrate. (a)** Photograph of ZnO NW arrays on a paper substrate**,** which maintained its flexibility after the ZnO NW growth. **(b)** Top-view, **(c)** tilted-view, and **(d)** enlarged SEM images of ZnO NW arrays on a paper substrate.

**Figure 4:**
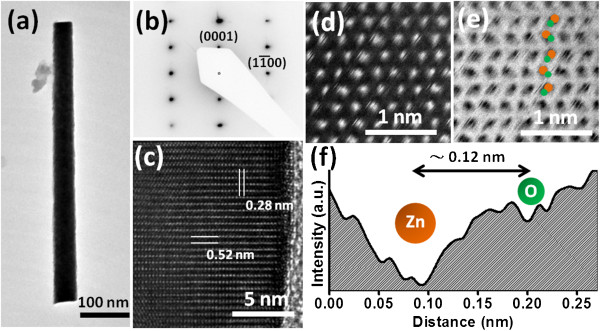
**TEM analysis of a hydrothermally grown ZnO NW. (a)** Low-magnification TEM image, **(b)** SAD pattern and **(c)** HR TEM image of a ZnO NW. **(d)** ADF and **(e)** ABF STEM images of a ZnO NW; the ABF STEM image reveals the ABAB stacking of the atoms in the ZnO. **(f)** ABF intensity profile across the Zn-O dumbbell pair in **(e)**.

Figure 3a displays a photograph of the paper sample after growth of the ZnO NWs; it retained the high flexibility required for potential applications in flexible devices. Figure 3b,c,d presents low-magnification top-view, tilted-view, and high-magnification SEM images, respectively. Notably, the ZnO NW arrays exhibited not only conformal coverage on the paper but also uniform distributions of their lengths and diameters. The average length and diameter of ZnO NWs on paper are 1.45 μm and 133 nm, respectively. From XRD analysis of ZnO NWs on paper, besides the signals from the paper substrate, other diffraction peaks correspond to wurtzite-structured ZnO (Additional file 1: Figure S4). Further, we used TEM to determine the crystal structure of a ZnO NW. Figure 4a displays a low-magnification TEM image of a ZnO NW; the SAD pattern in Figure 4b indicates that the NW was single-crystalline wurtzite-ZnO. The HR TEM image of the ZnO NW reveals (Figure 4c) atomic spacings of 0.28 and 0.52 nm, representing the (1-100) and (0001) planes of ZnO, respectively; thus, the ZnO NWs must have grown along the *c* axis. Structural analysis and the atomic arrangements of materials can also be resolved using aberration-corrected STEM analysis, a novel technique that provides visualized atomic arrangement information [23], especially when determining the polarity of wurtzite-structured compounds [24,25]. Figure 4d,e presents the high-angle-annular dark-field (HAADF) and corresponding annular-bright-field (ABF) HR STEM images, respectively, of a ZnO NW. In the HAADF HR STEM image, we could not resolve the dumbbell pairs of ZnO because of the low Z contrast of O atoms in HAADF imaging; in contrast, the ABF HR STEM image reveals dumbbell pairs of Zn and O atoms, with the O atoms (lower contrast) laying beside Zn atoms (higher contrast) in dumbbell pairs zigzagging along the *c* axis, exhibiting the ABAB stacking order of wurtzite-structured ZnO. From the intensity profile of the Zn-O dumbbell pairs (Figure 4f), we estimated the spacing between the Zn and O atoms to be 0.12 nm, consistent with the value reported previously [26]. Notably, the polarity of the hydrothermally grown ZnO NWs could be determined for the first time by STEM; because the O atoms were positioned just below the Zn atoms in the dumbbell pairs, the ZnO NWs possessed Zn polarity.

**Figure 5:**
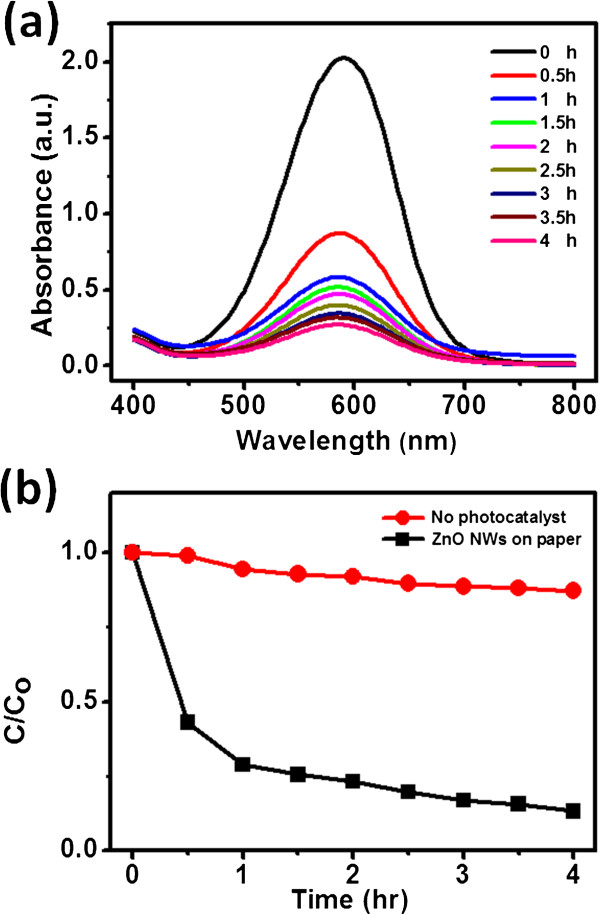
**Absorption spectra of MB in the presence of ZnO NW arrays. (a)** Absorption spectra of MB in the presence of ZnO NW arrays on a paper substrate under UV light illumination for different periods of time. **(b)** Photodegradation of MB in the presence and absence of ZnO NW arrays, plotted with respect to time.

We used the photodegradation of MB to evaluate the photocatalytic activity of our ZnO NW arrays on paper substrate. Samples of the same size (1 cm^2^) were first immersed in a 114 μM MB solution with continuous stirring and then exposed to light from a UV lamp along the normal direction. The wavelength of the UV lamp is 325 nm, which is shorter than the absorption edge (approximately 370 nm) of ZnO NWs on paper (Additional file 1: Figure S5), thus the electrons in ZnO can be excited to degrade the MB under UV lamp irradiation during the measurements. Figure 5a displays the absorption spectra of the MB solution in the presence of ZnO NWs on paper under exposure to UV light for various periods of time; the sample was grown using ZnO NPs from recipe F. The intensity of absorbance peak at 590 nm, which is typically proportional to the concentration of MB, decreased upon increasing the UV exposure time, confirming the decomposition of MB. For comparison, we performed an experiment in which we monitored the concentration of MB in a similar solution in the absence of the sample (ZnO NW arrays on paper substrate). We observed only a slight decrease in the concentration of MB (Figure 5b). Thus, we conclude that the ZnO NW arrays served as catalysts during the photodegradation process.

**Figure 6:**
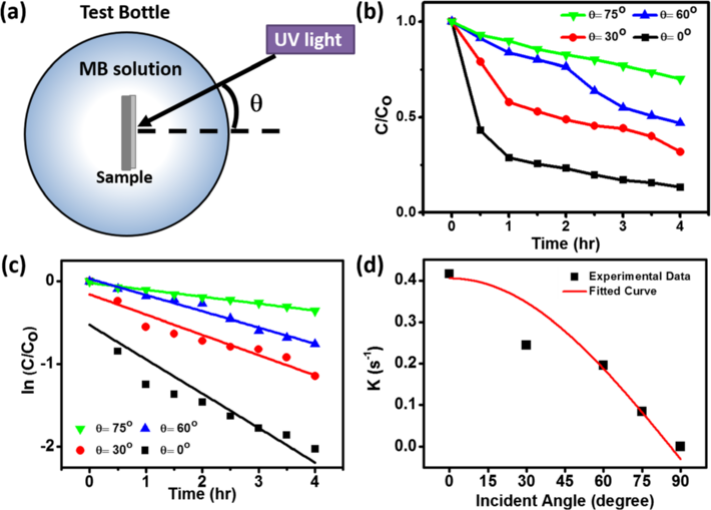
**Schematic representation of the setup and the angle-dependent photocatalytic activity of ZnO NW arrays on paper substrate. (a)** The figure illustrates the top-view of the setup showing the definition of incident angle in the present work. **(b)** Photodegradation of MB in the presence of ZnO NW arrays under illumination with UV light at incident angles of 0°, 30°, 60°, and 75°. **(c)** Data in (b) plotted on a logarithmic scale. **(d)** Extracted rate constants for reactions performed at various incident angles; the red curve is the fitted data, revealing that the rate constants were correlated to the cosine of the incident angle.

Next, we examined the influences of the incident angle of the UV light on the photocatalytic activity of the ZnO NW arrays on paper substrates. Figure 6a presents a schematic representation of the experimental setup; we define the incident angle (*θ*) as the angle between the illuminating UV light and the normal direction of the sample. The samples was immersed in a cylindrical test glass bottle for the experiments; the refraction of UV light at the air/solution interface can be regarded to be identical to different incident angles, because of the geometry of the glass bottle. Photodegradation experiments performed in the presence of ZnO NW arrays at different incident angles of UV light revealed (Figure 6b) that the degradation of MB was strongly dependent on the incident angle; the highest photocatalytic activity resulted when the UV light illuminated the sample from the normal direction (i.e., *θ* = 0°), with more than 86% of the MB degrading after 4 h. Because the degradation process can be regarded as having first-order reaction kinetics, the reaction can be described as

(1) $$C\left(t\right)=C\left(0\right)\;e^{–\text{kt}}\text{.}$$

This equation can be rewritten as

(2) $$\text{ln}\left[C\left(t\right)/C\left(0\right)\right]=–\text{kt},$$

where *C*, *k*, and *t* are the concentration of MB, the rate constant of the degradation process, and the reaction time, respectively. Figure 6c displays the data plotted on a logarithmic scale; the slope of each fitted line represents the rate constant of the degradation reaction performed at a particular angle of incident light. For incident angles of 0°, 30°, 60°, and 75°, the calculated rate constants were 0.415, 0.244, 0.195, and 0.08 s^-1^, respectively (Figure 6d). In its simplest model, the number of photogenerated electron/hole pairs involved in the photodegradation reactions would be proportional to the intensity of UV light. Here, the incident angle was the only parameter that we controlled. The intensity is typically correlated to the incident angle through the relationship.

(3) $$I=I_{0}\;\cos\;\theta \text{,}$$

where *I*_0_ is the intensity of light for illumination normal to the sample surface. When we fit the rate constants *k* as a function of the incident angle *θ* using Equation (3) (red curve in Figure 6d), the resulting curve fitted our data well.

## Conclusions

We have used a two-step growth strategy to prepare ZnO NW arrays on paper substrates. First, we synthesized ZnO NPs having a uniform size distribution (ca. 4 nm); here, the concentration of ZnO NPs was highly dependent on the amount of added NaOH. These ZnO NPs then served as seeds for the hydrothermal growth of ZnO NW arrays on paper substrates. When we used our solution containing the highest concentration of ZnO NPs, we obtained dense and uniform ZnO NW arrays that had grown conformally on the paper substrate. TEM analysis of these ZnO NWs not only revealed their single-crystalline nature but also confirmed the polarity of ZnO to be Zn-polarized (through ABF STEM analysis). From measurements of the photocatalytic activity toward MB of the ZnO NW arrays on paper substrates at incident angles of 0°, 30°, 60°, and 75°, we calculated rate constants of 0.415, 0.244, 0.195, and 0.08 s^-1^, respectively, revealing a dependence on the cosine of the incident angle of UV light. Such angle-dependent photocatalytic activity for biocompatible ZnO NWs on paper substrates suggests that such systems may have promising applications in the design of sterilization systems and light-harvesting devices.

## Abbreviations

ABF: annular-bright-field; HADDF: high-angle-annular dark-field; HMTA: hexamethylenetetramine; NP: nanoparticle; NW: nanowire; SEM: scanning electron microscopy; STEM: scanning transmission electron microscopy; TEM: transmission electron microscopy; XRD: X-ray diffractometry.

## Competing interests

The authors declare that they have no competing interests.

## Authors’ contributions

MY, YT, and CY designed this work, carried out the experiments, analyzed the results, and discussed the manuscript during the preparation. MY and CY prepared the manuscript. All authors reviewed the manuscript. All authors read and approved the final manuscript.

## Supplementary Material

Additional file 1 **Supplementary information Figure S1 (a) The SEM image and (b) the EDS spectrum of bare paper substrate.** The Pt signal is from the Pt thin layer which serves as the conducting layer for SEM observation. **Figure S2** The plot of pH values as a function of NaOH adding amount in ZnO nanoparticle formation solution. **Figure S3** (a) to (e) SEM images of ZnO nanowire arrays on paper substrate using the ZnO nanoparticles synthesized from recipes A,C,D,E and F, respectively. **Figure S4** XRD pattern of ZnO NWs on paper. Besides the signals from paper substrate (CaCO3 and Cellulose), all the diffraction peaks correspond to the wurtzite structured ZnO. **Figure S5** The absorbance of ZnO NWs on paper. Click here for file
