# Supplementary material for: Angle-dependent photodegradation over ZnO nanowire arrays on flexible paper substrates
Source: Nanoscale Res Lett. 2014 Dec 11;9(1):667. doi: 10.1186/1556-276X-9-667 (PMC4273692; doi:10.1186/1556-276X-9-667)
Supplement: Additional file 1 — Supplementary information Figure S1 (a) The SEM image and (b) the EDS spectrum of bare paper substrate. The Pt signal is from the Pt thin layer which serves as the conducting layer for SEM observation. Figure S2 The plot of pH values as a function of NaOH adding amount in ZnO nanoparticle formation solution. Figure S3 (a) to (e) SEM images of ZnO nanowire arrays on paper substrate using the ZnO nanoparticles synthesized from recipes A,C,D,E and F, respectively. Figure S4 XRD pattern of ZnO NWs on paper. Besides the signals from paper substrate (CaCO3 and Cellulose), all the diffraction peaks correspond to the wurtzite structured ZnO. Figure S5 The absorbance of ZnO NWs on paper. [file 1556-276X-9-667-S1.pdf]

Supporting Information

Angle-dependent photodegradation over ZnO nanowire arrays on flexible paper substrates

Ming-Yen Lu[1,2,*], Yen-Ti Tseng[1], and Cheng-Yao Chiu[1]

[1] Graduate Institute of Opto-Mechatronics, National Chung Cheng University, Chia-yi 62102, Taiwan, Republic of China

[2] Advanced Institute of Manufacturing with High-tech Innovations, National Chung Cheng University, Chia-Yi 62102, Taiwan

*E-mail: mylu@ccu.edu.tw

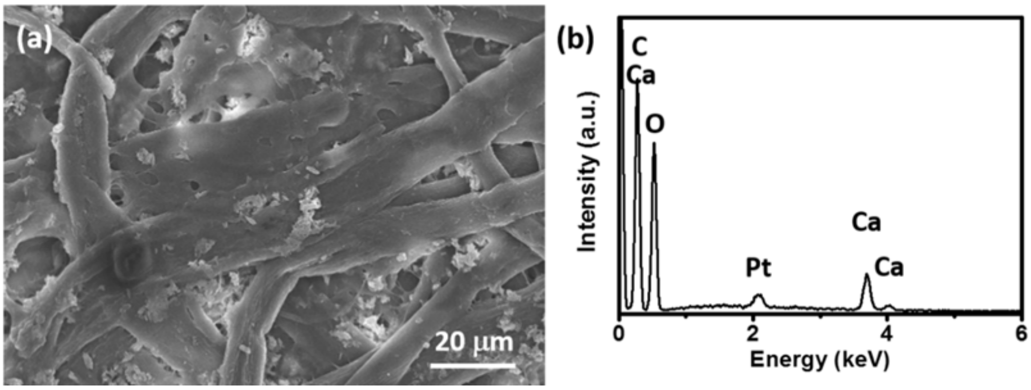

Figure S1 (a) The SEM image and (b) the EDS spectrum of bare paper substrate. The Pt signal is from the Pt thin layer which serves as the conducting layer for SEM observation.

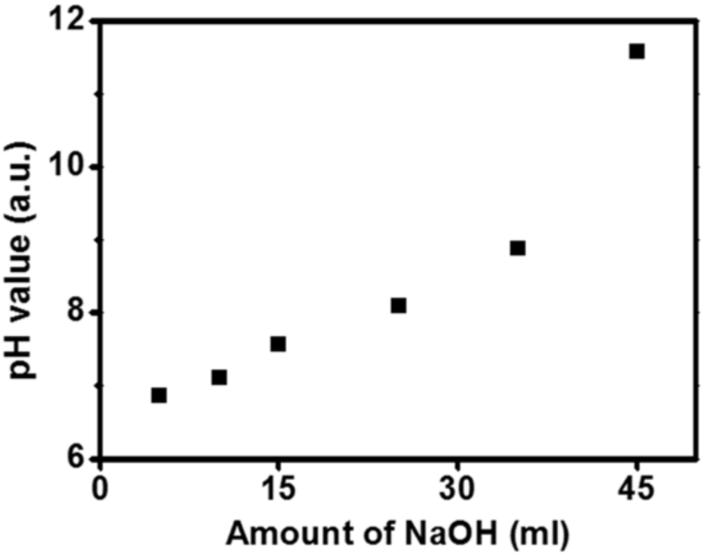

Figure S2 The plot of pH values as a function of NaOH adding amount in ZnO nanoparticle formation solution.

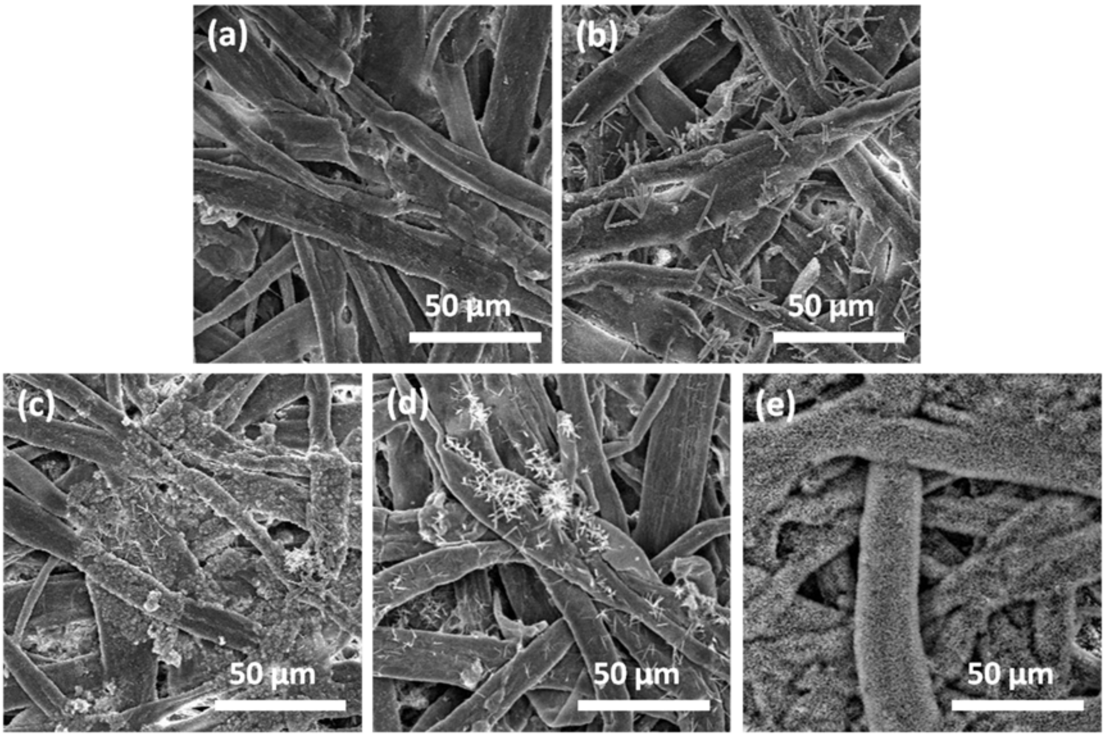

Figure S3 (a) to (e) SEM images of ZnO nanowire arrays on paper substrate using the ZnO nanoparticles synthesized from recipes A,C,D,E and F, respectively.

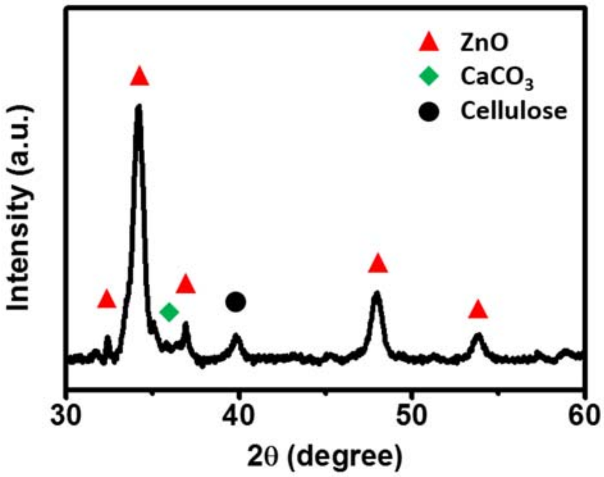

Figure S4 XRD pattern of ZnO NWs on paper. Besides the signals from paper substrate ($CaCO_3$ and Cellulose), all the diffraction peaks correspond to the wurtzite structured ZnO.

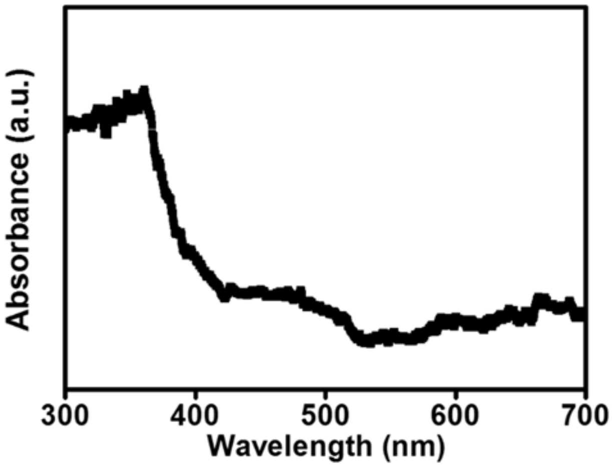

Figure S5 The absorbance of ZnO NWs on paper.
